# Lower Adherence to Breastfeeding Recommendations in Mothers Treated With Antirheumatic and Antidepressant Medications

**DOI:** 10.1177/08903344251337384

**Published:** 2025-05-27

**Authors:** Essi Whaites Heinonen, Diana L. Johnson, Alec Todd, Christina D. Chambers

**Affiliations:** 1 Center for Better Beginnings, Department of Pediatrics, University of California San Diego, La Jolla, CA, USA; 2 Division of Pediatrics, Department of Clinical Science, Intervention and Technology, Karolinska Institutet, Stockholm, Sweden; 3 Herbert Wertheim School of Public Health and Human Longevity Science, University of California San Diego, La Jolla, CA, USA

**Keywords:** antidepressants, antirheumatic medications, asthma medications, breastfeeding, breastfeeding barriers, breastfeeding initiation, disease-modifying antirheumatic drugs, lactation, prospective cohort study, selective serotonin reuptake inhibitors

## Abstract

**Background:**

Exclusive breastfeeding for 6 months is recommended, but breastfeeding safety data is insufficient for several medications.

**Aim:**

To determine if mothers treated with chronic medications are less likely to breastfeed.

**Methods:**

For this secondary analysis, 6383 pregnant women in the MotherToBaby cohort recruited from the United States and Canada between 2010 and 2022 were included. Participants treated with antirheumatic medications (ARM), selective serotonin reuptake inhibitors (SSRIs), and asthma medications during pregnancy were divided into two groups based on their medication use: continuers and discontinuers. Breastfeeding initiation, supplementation with commercial milk formula, and discontinuation of breastfeeding before 6 months were compared between those exposed and unexposed to medication use. Adjusted risk and hazard ratios (aRR, aHR) and 95% Confidence Intervals (CI) were calculated with modified Poisson and Cox regressions adjusted for year, parity, socioeconomic status, body mass index, smoking, illicit drug use, race and ethnicity.

**Results:**

The sample included 799 (12.5%) continuers and 475 (7.4%) discontinuers of ARM, 293 continuers (4.6%) and 63 (1.0%) discontinuers of SSRIs, and 217 (3.4%) continuers and 97 (1.5%) discontinuers of asthma medications. There were 4,439 (69.6%) participants who were unexposed to the study medications. Both ARM continuers and discontinuers were more likely to not breastfeed (aRRs 95% CI: 3.92 [3.03, 5.07] and 3.08 [2.19, 4.33]), to supplement (aRRs 95% CI: 1.12 [1.01, 1.26] and 1.25 [1.10, 1.43]) and stop breastfeeding before 6 months (aHRs 95% CI: 1.72 [1.29, 2.31] and 1.41 [0.92, 2.15]). SSRI continuers were more likely to supplement (aRR 95% CI: 1.26 [1.08, 1.47]).

**Conclusion:**

Participants treated with chronic medications, primarily ARMs, were less likely to breastfeed. Targeted lactation support for mothers with chronic illnesses is recommended along with development of breastfeeding safety data for these medications.

**Key Messages**

● Breastfeeding is the recommended sole source of nutrition for the first 6 months of life. For several chronic medications, there is a significant lack of safety data preventing mothers with chronic disorders from safely following this recommendation.

● Mothers treated with chronic antirheumatic medications including systemic steroids and immunomodulators during pregnancy were more likely to not initiate breastfeeding, to supplement with formula within 2 weeks from birth, and to stop breastfeeding before 6 months than untreated mothers.

● Mothers treated with selective serotonin reuptake inhibitors at the time of delivery were as likely as untreated mothers to initiate breastfeeding and to continue for 6 months but were more likely to supplement with formula.

● Early and targeted lactation support may be a way of improving the adherence to breastfeeding recommendations amongst women with chronic physical and psychiatric illnesses. Timely development and dissemination of safety data is needed for the newer antirheumatic medications that have recently entered the market.

Breastfeeding is known to have several health benefits for both mother and child, and is the recommended sole source of nutrition for the first 6 months of life (Meek & Noble, 2022). More than 70% of women who provide human milk take some medication, and more than 90% of medications lack appropriate labeling information for pregnant and lactating women (Ren et al., 2021). Most medications are likely to be safe to use during lactation, but the lack of definitive evidence leads to varying and uncertain recommendations on the safety of breastfeeding (Blattner et al., 2016; Byrne & Spong, 2019; Verstegen & Ito, 2019).

Most immunomodulators are considered safe to use during breastfeeding because of their low-risk pharmacokinetic properties, but studies on their safety are limited (Beltagy et al., 2021; Birru Talabi & Clowse, 2020). Even though selective serotonin reuptake inhibitor (SSRI) antidepressants are considered safe to use during lactation (Hale & Krutsch, 2023; Uguz, 2021), lower breastfeeding rates have been reported in women treated with them (Gorman et al., 2012). Inhaled asthma medications are expected to produce low medication levels in human milk and are therefore considered safe during lactation, but the data to support this are limited (Chambers et al., 2021; Fält et al., 2007).

Concerns regarding medication safety are a common reason to stop treatment with chronic medications during pregnancy. In a recent study funded by the U.S. Food and Drug Administration (FDA), 35% of pregnant women stopped their medication during pregnancy, a majority of them due to fears for infant safety (Head et al., 2023). While the reasons behind choosing not to breastfeed are individual and have not been well studied, being treated with chronic medications may have a negative impact on the decision to breastfeed, even when safety data are available for that medication (Passier & Woestenberg, 2023; Saha et al., 2015).

The aim of this study was to determine if mothers treated in pregnancy with selected chronically-used medications were less likely to follow breastfeeding recommendations compared to untreated mothers, and if these behaviors varied by whether or not the mother discontinued her medication earlier in pregnancy.

## Method

### Research Design

This was a secondary analysis performed using data from a prospectively collected observational cohort study. The study design was appropriate for capturing specific maternal medication use during pregnancy and near the time of delivery when initial breastfeeding decisions must be made, and for linking those data to subsequent breastfeeding decisions in the first 6-months postpartum. Ethics committee approval under IRB# 130658, approved December 26, 2023, was obtained from the Institutional Review Board at University of California San Diego and study participants provided informed consent.

### Setting and Relevant Context

The MotherToBaby cohort is open for enrollment for pregnant persons in the United States and Canada. The study design and methods have been described in detail elsewhere (Chambers et al., 2001, 2013, 1996). Briefly, the pregnant persons are referred or self-refer into the single site research center during pregnancy and are followed to pregnancy outcome. Liveborn infants are additionally followed to 1 year after birth. The persons were considered eligible to enroll in the study if they resided in the United States or Canada, spoke English or Spanish, did not have prior knowledge of the outcome of the pregnancy, and agreed to the study protocol. Exposure, outcome, and covariate information were obtained through repeated telephone interviews and confirmed by medical records when available. For this secondary data analysis, participants were selected who had previously enrolled in various MotherToBaby sub studies, a few of which involved compensation for engaging in the original study.

For context regarding breastfeeding as an outcome, the breastfeeding initiation rate in the United States is around 83%, and around 90% in Canada, whereas the breastfeeding rate at 6 months is around 60% in both countries (*Breastfeeding Among U.S. Children Born 2013–2020*, 2023; *Canada’s Breastfeeding Progress Report*, 2022). In these countries, breastfeeding is supported by lactation specialists and other health care professionals, with the goal to increase the rate of 6-month exclusive breastfeeding. However, in the United States, breastfeeding is often complicated by short parental leaves, around 8–12 weeks, if any. Vulnerable populations for not breastfeeding and for early breastfeeding cessation in both United States and Canada are persons with low socioeconomic status, obese persons, and ethnic minorities (*Canada’s Breastfeeding Progress Report*, 2022; Meek & Noble, 2022).

### Sample

**Figure 1.**
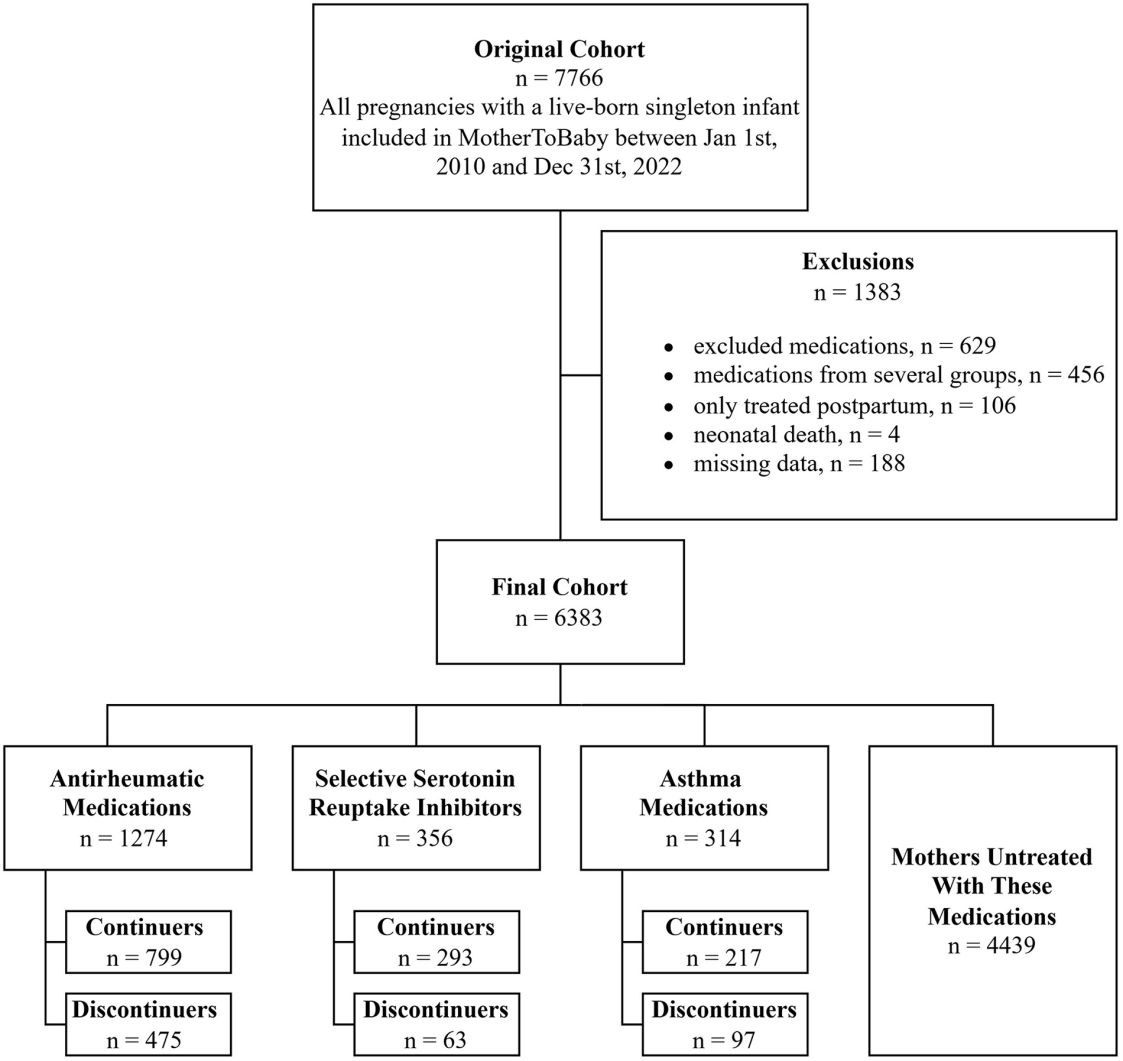
Flowchart of the Study Cohort. *Note.* SSRIs = selective serotonin reuptake inhibitors.

The target population consisted of all breastfeeding persons in the United States and Canada who were eligible to enroll in the MotherToBaby Pregnancy Studies cohort. For this secondary analysis, the initial selection included 7,766 pregnant women enrolled in the MotherToBaby-cohort during the study period 2010–2022, who delivered a singleton, live-born infant. Exclusions were applied for women who were treated with neurotropic medications other than SSRIs (*n* = 570) and immunomodulators recommended not to be used during lactation by the Drugs and Lactation Database (Lactmed; *n* = 71), as well as mothers treated with medications from more than one exposure group (*n* = 456), neonatal death (*n* = 4), mothers only treated postpartum (*n* = 106), or missing data on breastfeeding (*n* = 188; Figure 1, Supplementary Table 1; National Institute of Child Health and Human Development, 2006). After these exclusions, the final study cohort consisted of 6383 participants. The cohort consisted of four groups:

1. Participants treated with ARMs (*n* = 1274, 20%), of whom 799 (63%) continued use throughout pregnancy and 475 (37%) discontinued use earlier in pregnancy; (2) participants treated with SSRIs (*n* = 356, 6%), of whom 293 (82%) were continuers and 63 (18%) discontinuers; (3) participants treated with asthma medications (*n* = 314, 5%), of whom 217 (69%) were continuers and 97 (31%) discontinuers; and (4) participants not treated with any of the included medications (*n* = 4439, 70%) (Figure 1).

The overall MotherToBaby cohort is an open cohort study with no a priori determined sample size for the different exposures. For this secondary breastfeeding analysis, a power analysis with the available sample sizes showed that we had 80% power to rule out a 2-fold increased risk for not initiating breastfeeding for continuers and discontinuers of antirheumatic medications, but were less well-powered for the other exposure groups.

### Measurement

#### Exposures

The exposure groups were defined as use of the included medications during pregnancy and at the time of delivery for continuers, and during pregnancy but not at the time of delivery in discontinuers. The exposed participants could be treated with more than one medication within the same exposure group. The untreated comparison group was not exposed to any of these studied medications during pregnancy or within 3 months postpartum. The most common medications in the ARM group were prednisone, adalimumab, and hydroxychloroquine. Those most common in the SSRI-group were sertraline and escitalopram. In the asthma medication group, fluticasone propionate and budesonide, alone or in combination with salmeterol or formoterol fumarate, were most commonly reported (see Supplementary Table 2 in the online supplemental material).

#### Outcomes

Not initiating breastfeeding was defined as never breastfed or given mother’s own milk. Participants who had initiated breastfeeding were asked whether they had breastfed exclusively or supplemented with formula in the first 2 weeks after birth. Cessation of breastfeeding before 6 months was defined as mother reporting a stop date for breastfeeding that was before 6 months postpartum. Potential sources of bias were addressed by using standard interview protocol for obtaining exposure and outcome data and information on covariates, captured by trained interviewers.

#### Covariates

Pre-pregnancy body mass index (BMI) measured in kg/m^2^ (categories < 18.5 / 20–24.9 / 25–29.9 / 30–34.9 / > 35) and the participant’s age in years (categories < 20 / 20–35 / > 35) were presented as categorical variables (Table 1). Socioeconomic status (SES) was calculated at the time of enrollment with the Hollingshead 4-factor score based on maternal and paternal occupation and education (Adams & Weakliem, 2011). These SES scores were categorized in rank order 1–5 with 1 being the highest. These were recoded to a binary variable (scores 1–3 vs. 4–5). Year of delivery (2010–2016 vs. 2017–2022), and parity (primipara/multipara) were recoded to binary variables (Table 1). Exposures to alcohol, tobacco, and recreational drugs were defined as exposure reported any time during the pregnancy and presented as binary variables (yes/no).

**Table 1.** Background Characteristics of the Included Mother–Infant Pairs, *N* = 6383.

Characteristic	ARMs		SSRIs		Asthma Medications		Untreated	Difference Between Groups	
	Cont. *n* = 799	Discont. *n* = 475	Cont. *n* = 293	Discont. *n* = 63	Cont. *n* = 217	Discont. *n* = 97	*n* = 4439	*X* ^2^	*p*
	*n* (%)	*n* (%)	*n* (%)	*n* (%)	*n* (%)	*n* (%)	*n* (%)		
Year of Delivery								159.25	< 0.001
2010–2016	469 (59)	298 (63)	73 (25)	24 (38)	114 (53)	51 (53)	2006 (45)		
2017–2022	330 (41)	117 (37)	220 (75)	39 (62)	103 (47)	46 (47)	2433 (55)		
Age, Years								14.30	0.282
< 20	5 (1)	2 (0)	0 (0)	0 (0)	2 (1)	2 (2)	21 (1)		
20–35	623 (78)	355 (75)	217 (75)	50 (79)	170 (78)	67 (69)	3416 (77)		
> 35	171 (21)	118 (27)	76 (26)	13 (21)	45 (21)	28 (29)	999 (23)		
Primipara	431 (54)	267 (56)	129 (44)	30 (48)	127 (59)	59 (61)	2148 (48)	32.02	< 0.001
Pre-Pregnancy BMI, kg/m^2^								49.10	0.002
< 18.5	37 (5)	15 (3)	6 (2)	0 (0)	7 (3)	2 (2)	130 (3)		
18.5–24.9	448 (56)	250 (53)	145 (50)	36 (57)	94 (44)	55 (57)	2556 (58)		
25–29.9	181 (23)	127 (27)	72 (25)	14 (22)	63 (29)	21 (22)	1013 (23)		
30–34.9	75 (9)	49 (10)	41 (14)	7 (11)	27 (13)	9 (9)	430 (10)		
30–34.9	55 (7)	33 (7)	27 (9)	6 (10)	25 (12)	10 (10)	274 (6)		
≥ 35	37 (5)	15 (3)	6 (2)	0 (0)	7 (3)	2 (2)	130 (3)		
Ethnicity Hispanic / Latina	74 (10)	43 (9)	19 (7)	3 (5)	19 (9)	5 (5)	599 (14)	35.15	< 0.001
Race								55.42	< 0.001
White	693 (88)	398 (87)	271 (94)	54 (87)	178 (83)	76 (78)	3524 (82)		
Black	25 (3)	21 (5)	5 (2)	1 (2)	12 (6)	7 (7)	219 (5)		
Asian	30 (4)	20 (4)	6 (2)	3 (5)	10 (5)	6 (6)	274 (6)		
Other ^a^	29 (4)	16 (4)	6 (2)	4 (7)	11 (5)	6 (6)	193 (5)		
SES ^b^								24.68	< 0.001
1–3	730 (91)	434 (91)	247 (84)	46 (73)	198 (91)	93 (96)	3670 (84)		
4–5	40 (5)	25 (5)	12 (4)	4 (6)	14 (7)	2 (2)	353 (8)		
Smoking ^c^	22 (3)	27 (6)	11 (4)	5 (8)	6 (3)	7 (7)	145 (3)	16.82	0.010
Alcohol ^c^	361 (45)	220 (46)	144 (49)	29 (46)	102 (47)	52 (54)	2055 (46)	3.44	0.752
Illicit drugs ^c^	19 (2)	17 (4)	16 (6)	5 (8)	7 (3)	5 (5)	109 (3)	19.50	0.003
Infant Assigned Female at Birth	380 (48)	228 (48)	130 (45)	38 (60)	103 (48)	46 (47)	2201 (50)	7.06	0.315
Cesarean Section	321 (40)	184 (39)	91 (31)	17 (27)	79 (36)	28 (29)	1188 (27)	83.87	< 0.001
Preterm Birth	93 (12)	33 (7)	24 (8)	1 (2)	14 (7)	5 (5)	215 (5)	60.90	< 0.001
APGAR-Score 5’ < 7	11 (2)	1 (0)	9 (3)	1 (2)	3 (1)	2 (2)	44 (1)	16.59	0.011
NICU	94 (12)	53 (11)	39 (13)	5 (8)	20 (9)	12 (12)	412 (9)	10.46	0.107

*Note.* ARMs = Antirheumatic Medications, Cont. = Continuer, Discont. = Discontinuer, SSRIs = Selective Serotonin Reuptake Inhibitors, BMI = Body Mass Index, SES = Socioeconomic Scale, NICU = Neonatal Intensive Care Unit admission, *X*^2^ = Value for Pearson Chi-Square, *p* = two-sided *p* value between exposure groups calculated with Chi-Square, with *p* < 0.05 considered statistically significant. Missing values: Age = 3, Primipara = 1, Body Mass Index = 43, Ethnicity = 49, Race = 155, SES = 515, Infant sex = 20, Cesarean sectio*n* = 3, APGAR-score 5 min < 7 = 1479, NICU = 19.

a Pacific islander, Indian/Native American, Other.

b Calculated with Hollingshead Four-Factor scale.

c Recorded any time during pregnancy.

### Data collection

All data were collected from the MotherToBaby cohort, which enrolled women in a prospective study during their current pregnancies, between January 1, 2010, and December 31, 2022. Informed consent was collected from all participants at inclusion. All participants were enrolled during pregnancy before knowledge of birth or breastfeeding outcomes, and data were collected by trained study interviewers and data abstractors. At the time of enrollment, the women completed a telephone interview covering demographics, reproductive history, maternal health including pre-pregnancy BMI and exposures during the current pregnancy. The exposures were then assessed repeatedly during follow-up interviews throughout the pregnancy. The exposure history included dosages, dates, and indications for medications and use of recreational substances including alcohol and tobacco. Medical records were also abstracted from the obstetrician, pediatrician, any specialty physician, and hospital of delivery.

Information on breastfeeding initiation and supplementation within 2 weeks after birth was recorded in the first postpartum telephone interview conducted typically in the 6 weeks after delivery. Data on breastfeeding duration was collected at repeated telephone interviews at 3 and 6 months postpartum, and an estimated date of when the breastfeeding was discontinued was documented, when available. The data were stored in the study database.

### Data Analysis

Background characteristics of the study population and frequencies of the outcomes of not initiating breastfeeding and supplementing with formula were presented as numbers and percentages by exposure group. Differences between groups were compared using Pearson’s Chi square and Fisher’s exact tests with two-tailed *p* values to judge significance. Adjusted risk ratios (aRRs) and their 95% Confidence Intervals (CIs) for not initiating breastfeeding and for supplementing with formula were calculated using modified Poisson regression analyses with robust covariances. These analyses were adjusted for a propensity score composed of the confounders that were selected a priori by the investigators as being relevant: parity, pre-pregnancy BMI, year of delivery, socioeconomic status, smoking, illicit drug use, race, and ethnicity. This method was chosen in favor of logistic regression, as odds ratios were expected to overestimate the risks. Preterm birth, Cesarean section and neonatal morbidity were considered as potential mediators rather than true confounders for adverse breastfeeding outcomes, and were therefore not adjusted for in the analyses. Therefore, the observed effect estimates include any mediation through the chosen delivery method and perinatal morbidity.

Outcome data on breastfeeding duration was truncated at 6 months, consistent with the breastfeeding recommendations. Data from women without known stop dates for breastfeeding were censored at the time of the latest documented interview when the mother was still breastfeeding. Kaplan Meier survival curves for breastfeeding duration within 6 months were calculated for the different exposure groups, and adjusted hazard ratios (aHRs) and 95% CIs of stopping breastfeeding within 6 months were compared between groups with Cox regression adjusted for a propensity score composed of the same relevant covariates as for other outcomes. Indications for goodness of fit for the regression analyses are presented in Supplementary Tables 3 and 4. Missing data on included covariates resulted in exclusion from the propensity-score adjusted analyses and are presented in Table 1. Statistical analyses were performed with SPSS (Version 29.0).

## Results

### Characteristics of the Study Population

Demographic factors, maternal characteristics and birth characteristics of the 6,383 included mother–infant dyads are presented in Table 1. Included and excluded maternal medications are presented in Supplementary Tables 1 and 2. Women in all exposure groups had similar ages but differed significantly regarding year of delivery (*X*^2^ = 159.25, *p* < 0.001), socioeconomic status (*X*^2^ = 24.68, *p* < 0.001) and pre-pregnancy BMI (*X*^2^ = 49.10, *p* = 0.002). The rate of alcohol use was similar between the groups, but discontinuers of all medications were more likely to have smoked during pregnancy than continuers and the untreated participants (*X*^2^ = 16.82, *p* = 0.010). Women treated with SSRIs, both continuers and discontinuers, had a significantly higher rates of substance use than the unexposed (*X*^2^ = 19.50, *p* = 0.003), mainly consisting of use of cannabinoid products. Around half of all participants were multiparas, but women treated with ARMs and asthma medications during pregnancy, both continuers and discontinuers, were more likely to report that the child was their first, compared to women treated with SSRIs and the unexposed women (*X*^2^ = 32.02, *p* < 0.001). Of all participants, 5,194 (83%) identified as White, with a slightly larger proportion in the ARM and SSRI groups, both continuers and discontinuers, than the asthma and unexposed groups (*X*^2^ = 55.42, *p* < 0.001). The highest rates of Cesarean sections and preterm birth were found amongst continuers of ARMs (*X*^2^ = 83.87, *p* < 0.001 and *X*^2^ = 60.90, *p* < 0.001, respectively), whereas the infants to continuers of SSRIs had a slightly higher rate of low APGAR-scores than infants to participants in the other groups (*X*^2^ = 16.59, *p* = 0.011).

### Breastfeeding Outcomes

Data on breastfeeding initiation were available for all the 6,383 included participants, and on supplementing with for 5,198 (98%) of the 6,071 participants who initiated breastfeeding. Of the participants who were breastfeeding, 5,598 (87.7%) answered a breastfeeding questionnaire after the initial 14 days postpartum, and 1,591 (26%) after 6 months postpartum. Of all the included participants, 312 (5%) did not initiate breastfeeding, and out of the 6,071 (95%) participants who did initiate breastfeeding, 2,070 (35%) supplemented with commercial milk formula within 2 weeks after birth.

**Figure 2.**
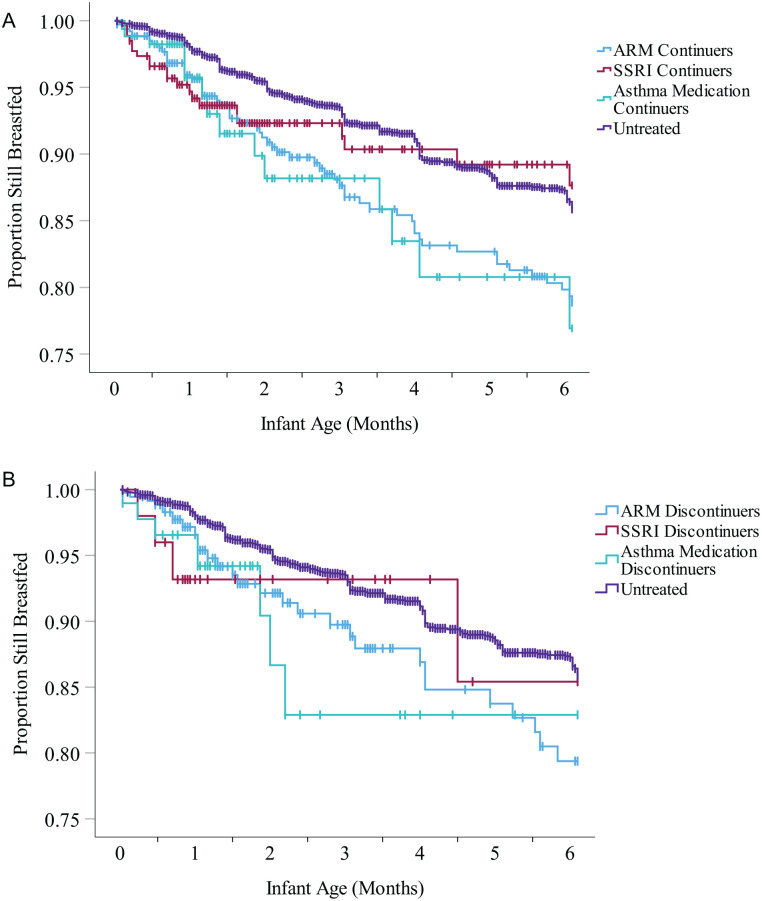
Survival Curves for Breastfeeding Cessation Rates in the Different Exposure Groups Amongst Treatment Continuers (A) and Discontinuers (B) in the First 6 Months Postpartum. *Note.* The proportion still breastfed amongst women who initiated breastfeeding. Log-rank test *p* value 0.001 for continuers (A) and 0.060 for discontinuers (B). ARM = antirheumatic medications, SSRI = selective serotonin reuptake inhibitors, | = Censored case.

Out of the 799 continuers and 475 discontinuers of ARMs, 104 (13%) and 50 (11%) did not initiate breastfeeding, compared to 113 (3%) of the 4,439 untreated participants (*X*^2^ = 157.38, *p* < 0.001 and *X*^2^ = 67.86, *p* < 0.001, respectively; Table 2). Adjusted risk estimates for not initiating breastfeeding were 3.92 (95% CI [3.03, 5.07]) and 3.08 (95% CI [2.19, 4.33]), respectively (Table 3; Supplementary Table 3). Out of the 695 continuers and 425 discontinuers of ARMs who initiated breastfeeding, 258 (38%) and 174 (42%) supplemented with formula within 2 weeks from delivery, compared to 1,392 (33%) of the 4,306 untreated participants who initiated breastfeeding (*X*^2^ = 4.56, *p* = 0.033 and *X*^2^ = 12.12, *p* < 0.001). Adjusted risk estimates for supplementing were 1.12 (95 % CI [1.01, 1.26]) and 1.25 (95% CI [1.10, 1.43]), respectively. The hazard of stopping breastfeeding before 6 months was increased for both groups; however, after adjustment, it remained statistically significant only among the continuers, adjusted HR (95% CI 1.72 [1.29, 2.31]), (Table 3, Supplementary Table 4, Figure 2).

**Table 2.** Percentages of Participants Treated at Delivery and Treatment Discontinuers Not Initiating Breastfeeding and Supplementing With Formula Within the First 2 Weeks.

	Not Initiating Breastfeeding			Supplementing With Formula		
Exposure Group	*n*/*N* (%)	*X* ^2^	*p*	*n*/*N*	*X* ^2^	*p*
ARM continuers	104 / 799 (13%)	157.38	< 0.001	258 / 688 (38%)	4.56	0.033
ARM discontinuers	50 / 475 (11%)	67.86	< 0.001	174 / 416 (42%)	12.12	< 0.001
SSRI continuers	9 / 293 (3%)	0.005	0.860	121 / 283 (43%)	10.48	0.001
SSRI discontinuers	4 / 63 (6%)	2.37	0.124	16 / 58 (28%)	0.85	0.402
Asthma medication continuers	12 / 217 (6%)	4.40	0.044	75 / 203 (37%)	1.13	0.287
Asthma medication discontinuers	0 / 97 (0%)	2.99	0.118	34 / 95 (36%)	0.25	0.660
Untreated	133 / 4439 (3%)	reference	reference	1392 / 4175 (33%)	reference	reference

*Note.* ARM = Antirheumatic medications, SSRI = Selective serotonin reuptake inhibitors. Treatment discontinuers were participants who were treated with the included drugs during pregnancy but not at the time of delivery. *X*^2^ = test value for Pearson Chi-Square, *p* = two-sided *p* values compared to untreated, calculated with Fisher’s exact test, with *p* < 0.05 considered statistically significant.

ARMs.

#### SSRIs

Out of the 293 continuers and 63 discontinuers of SSRIs, nine (3%) and four (6%) did not initiate breastfeeding, and the risk for not initiating breastfeeding was not significantly increased for either when compared to the untreated participants (Tables 2 and 3; Supplementary Tables 3 and 4). Out of the 284 continuers and 59 discontinuers of SSRIs who initiated breastfeeding, 121 (43%) and 16 (28%) supplemented with formula within 2 weeks from delivery (*X*^2^ = 10.48, *p* = 0.001 and *X*^2^ = 0.85, *p* = 0.402 when compared to untreated). The adjusted risk for supplementing with formula was increased for continuers (aRR 95% CI 1.26 [1.08, 1.47]), but not discontinuers. The hazard of stopping breastfeeding before 6 months was not significantly increased for either of the groups (Table 3, Figure 2).

**Table 3.** Risk Estimates for not Initiating Breastfeeding, Supplementing in the First 2 Weeks, and Stopping Breastfeeding Before 6 Months, When Compared to the Unexposed, for Participants Treated at Delivery and Participants who Discontinued Treatment During Pregnancy.

	Risk of Not Initiating Breastfeeding		Risk of Supplementing With Formula		Hazard of Stopping Before 6 Months	
Exposure Group	Crude RR [95% CI]	Adj. RR [95% CI]	Crude RR [95% CI]	Adj. RR [95% CI]	Crude HR [95% CI]	Adj. HR [95% CI]
ARM cont.	4.34 [3.40, 5.55]	3.92 [3.03, 5.07]	1.13 [1.01, 1.25]	1.12 [1.01, 1.26]	1.65 [1.24, 2.19]	1.72 [1.29, 2.31]
ARM disc.	3.51 [2.57, 4.80]	3.08 [2.19, 4.33]	1.26 [1.11, 1.42]	1.25 [1.10, 1.43]	1.55 [1.05, 2.30]	1.41 [0.92, 2.15]
SSRI cont.	1.03 [0.53, 1.99]	1.31 [0.65, 2.61]	1.28 [1.11, 1.48]	1.26 [1.08, 1.47]	1.22 [0.78, 1.90]	1.03 [0.63, 1.68]
SSRI disc.	2.12 [0.81, 5.55]	1.59 [0.56, 4.58]	0.83 [0.54, 1.26]	0.80 [0.49, 1.30]	1.45 [0.54, 3.89]	1.39 [0.44, 4.35]
Asthma med. cont.	1.85 [1.04, 3.28]	1.44 [0.74, 2.79]	1.11 [0.92, 1.33]	1.01 [0.84, 1.22]	1.85 [1.08, 3.18]	1.75 [1.02, 3.02]
Asthma med. disc.	NA	NA	1.07 [0.82, 1.41]	1.00 [0.76, 1.33]	1.83 [0.86, 3.88]	1.57 [0.70, 3.53]

*Note.* ARM = antirheumatic medications, SSRIs = selective serotonin reuptake inhibitors, med = medication, RR = risk ratio, HR = hazard ratio, Adj = Adjusted. Treatment continuers (cont.) were treated during pregnancy throughout the time of delivery, whereas discontinuers (disc.) were treated during pregnancy but discontinued treatment before the delivery. Models adjusted for a propensity score composed of parity, maternal pre-pregnancy body mass index, year of delivery, socioeconomic scale, smoking, illicit drug use, race, and ethnicity. Difference between groups was considered significant if the 95% CI did not cross 1.00.

#### Asthma medications

Out of the 217 continuers of asthma medications, 12 (6%) did not initiate breastfeeding (*X*^2^ = 4.40, *p* = 0.044 compared to untreated), but all of the 97 discontinuers did (Table 2). The crude risk for not initiating breastfeeding was increased for asthma medication continuers, but, after adjustments, this risk increase was no longer significant with 95% CI including 1.0. Out of the 205 continuers and 97 discontinuers of asthma medications who initiated breastfeeding, 75 (37%) and 34 (36%) supplemented with formula, similar to untreated participants. For the continuers of asthma medications, the adjusted hazard of stopping breastfeeding before 6 months was slightly increased, aHR 95% CI 1.75 [1.02, 3.02], but not for the discontinuers (Table 3; Supplementary Table 4; Figure 2).

## Discussion

This study found that participants treated with ARMs during pregnancy were more likely to not breastfeed, to supplement with formula and to stop breastfeeding before 6 months. We also found that continuers of treatment with SSRIs, but not discontinuers, were more likely to supplement with formula than the unexposed. For the participants treated with asthma medications, the crude risk estimates for not initiating breastfeeding and stopping before 6 months were elevated, but adjustments for covariates weakened these associations markedly.

To our knowledge, this is the largest cohort to date with prospectively collected data on breastfeeding outcomes in relation to chronically used maternal medications, and the first to present breastfeeding rates in mothers treated with ARMs during pregnancy and at the time of delivery. The increased risks for all three studied outcomes found in this study for participants treated with ARMs during pregnancy—both continuers and discontinuers—are concerning. This may in part be explained by the large number of ARMs that have entered the market without sufficient safety data for use during pregnancy and lactation in the last 2 decades (Birru Talabi & Clowse, 2020). The clinical consensus is that these medications are generally safe to use during lactation due to their low-risk pharmacological properties (Birru Talabi & Clowse, 2020), but as avoidant behavior—that is, fear of risks—has been associated with discontinuation of chronic medications during pregnancy (Head et al., 2023), similar cautionary attitudes may play a role in the decision not to breastfeed among mothers with chronic rheumatic disorders. This is supported by both continuers and discontinuers of antirheumatic medications being more likely to choose to not breastfeed.

However, as we did not know the reasons behind these choices, the lower breastfeeding rates we found in mothers treated with ARMs, which were not seen as clearly in mothers treated with SSRIs and asthma medications, could also reflect the severity of the underlying inflammatory disorder. The risk for not initiating breastfeeding was higher for continuers than discontinuers, even though the confidence intervals were overlapping, which might also suggest that the discontinuers were not as ill as the continuers, and therefore more likely to initiate breastfeeding.

The breastfeeding rates among participants treated with SSRIs in this analysis were markedly higher compared to a study on data from the MotherToBaby cohort published more than a decade ago (Gorman et al., 2012). This finding suggests that the numerous studies on use of SSRIs during lactation that have supported safety have been sufficiently disseminated (Uguz, 2021). However, participants treated with SSRIs at the time of delivery were still more likely to supplement with formula than untreated participants, indicating a further need for lactation support among these mothers.

The reasons for negative breastfeeding outcomes amongst mothers treated with ARMs and SSRIs are likely to be multifactorial, including consideration of the medication safety for the infant, the severity of the maternal disorder, socioeconomic factors, and perinatal complications. As mothers with chronic illnesses and medications have an increased risk for perinatal complications, this is also likely to be reflected in our results. We conclude that the breastfeeding decisions are always individual and multifactorial. Therefore, we recommend increased targeted lactation support to mothers with chronic illnesses, with or without chronic medications, to improve their adherence to the breastfeeding recommendations.

## Limitations

Being a secondary data-analysis on previously collected data, the interpretation of the results was limited by the lack of information on the reasons behind the breastfeeding decisions, as well as why treatment was discontinued during pregnancy. Also, reliable data was lacking on maternal medications during the breastfeeding period. Therefore, we do not know if these mothers restarted treatment or started a new treatment after delivery, which could have affected our measured outcomes, especially the breastfeeding duration. Future prospective studies should focus on the collection of in-depth data on the exposures at the different time points, as well as the reasons behind treatment discontinuation and the breastfeeding decisions. These studies are needed to know how to increase the percentage of mothers with chronic illnesses and medications breastfeeding exclusively for the recommended 6 months.

The interpretation of the breastfeeding outcomes for mothers treated with asthma medications was limited by sample size. However, as expected, none of the risks for this group were markedly increased. A limitation to the generalizability of our results was the higher breastfeeding rates in the MotherToBaby cohort than the national averages (*Breastfeeding Among U.S. Children Born 2013–2020*, 2023). All pregnant women in the United States and Canada were eligible to screen for participation in the MotherToBaby cohort, with a broad recruitment through social media, the MotherToBaby counseling service, and health-care providers. Due to the voluntary nature of participation, some level of healthy volunteer bias was likely to be present, but this bias should not have been differential between exposure groups.

In this study, we aimed to study the breastfeeding rates in women exposed to medications that were considered generally safe to use during lactation, and therefore excluded medications with unclear or contradictory recommendations. Most of the excluded neurotropic medications are considered safe to use during lactation, but due to the varying breastfeeding recommendations in the literature, they were excluded (Uguz, 2021). Also, for many of the included ARMs generally considered safe to use during lactation by professionals, the wordings of the breastfeeding recommendations varied, with the FDA-labels holding the most restrictive recommendations (Blattner et al., 2016; National Institute of Child Health and Human Development, 2006; Hale & Krutsch, 2023; Organization of Teratology Information Specialists (OTIS), 1994). Therefore, some misclassification might be present, depending on which recommendation source is followed. However, as these widely varying recommendations are also the reality for these patients and their health care providers, future attempts should be made towards unification of the recommendations.

## Conclusion

In summary, mothers treated with ARMs and SSRI antidepressants during pregnancy were less likely to breastfeed exclusively for the recommended 6 months than the untreated mothers. Specifically, both continuers and discontinuers of ARMs had increased risks of all the three studied breastfeeding outcomes, and SSRI continuers—but not discontinuers—were more likely to supplement with formula than mothers not treated with these chronic medications. As we did not know the reasons behind these decisions, and cannot fully distinguish the influence of the medication from the underlying disorder, we recommend targeted lactation support to all mothers treated with chronic medications before, during, and after pregnancy. Also, timely development and dissemination of lactation safety data for chronic medications is needed to better support this clinical decision-making.

## Supplemental Material

sj-docx-1-jhl-10.1177_08903344251337384 – Supplemental material for Lower Adherence to Breastfeeding Recommendations in Mothers Treated With Antirheumatic and Antidepressant Medications Supplemental material, sj-docx-1-jhl-10.1177_08903344251337384 for Lower Adherence to Breastfeeding Recommendations in Mothers Treated With Antirheumatic and Antidepressant Medications by Essi Whaites Heinonen, Diana L. Johnson, Alec Todd and Christina D. Chambers in Journal of Human Lactation
