# Supplementary material for: Lower Adherence to Breastfeeding Recommendations in Mothers Treated With Antirheumatic and Antidepressant Medications
Source: J Hum Lact. 2025 May 27;41(3):412–22. doi: 10.1177/08903344251337384 (PMC12238658; doi:10.1177/08903344251337384)
Supplement: sj-docx-1-jhl-10.1177_08903344251337384 – Supplemental material for Lower Adherence to Breastfeeding Recommendations in Mothers Treated With Antirheumatic and Antidepressant Medications [file sj-docx-1-jhl-10.1177_08903344251337384.docx]

# Lower Adherence to Breastfeeding Recommendations in Mothers Treated With Antirheumatic and Antidepressant Medications

**Supplementary Material**

**Supplementary table 1**

*Medications in Mothers Excluded Due to Exposure, N= 629*

| Medication Category | Excluded Medications |
| --- | --- |
| Neurotropic medications, n=570 | alprazolam, amitriptyline, aripiprazole, atomoxetine, bupropion, buspirone, carbamazepine, clomipramine, clonazepam, dextroamphetamine, diazepam, haloperidol, hydroxyzine, lamotrigine, lisdexamfetamine, lorazepam, methylphenidate, mirtazapine, olanzapine, oxcarbazepine, pregabalin, quetiapine, risperidone, venlafaxine, zaleplon, zolpidem, zopiclone |
| Antirheumatic medications, n=71 | leflunomide, methotrexate, mycophenolate mofetil, teriflunomide, tofacitinib, upadacitinib |

*Note:* Women exposed to the neurotropic and antirheumatic medications listed were excluded from all exposure groups.

**Supplementary Table 2**

*Medication Exposures in Mothers Treated With Antirheumatic Medications (N=1274), Selective Serotonin Reuptake Inhibitors (N=356) and Asthma Medications (N=314)*

| Antirheumatic Medications | | SSRIs | | Asthma Medications | |
| --- | --- | --- | --- | --- | --- |
| Medication | n (%) | Medication | n (%) | Medication | n (%) |
| Adalimumab | 368 (22) | Sertraline | 198 (56) | Fluticasone propionate / Salmeterol | 75 (25) |
| Prednisone | 333 (20) | Escitalopram | 74 (21) | Budesonide | 67 (22) |
| Hydroxychloroquine | 200 (12) | Fluoxetine | 45 (13) | Fluticasone propionate | 59 (19) |
| Certolizumab pegol | 200 (12) | Citalopram | 31 (9) | Budesonide / Formoterol fumarate | 55 (18) |
| Etanercept | 192 (12) | Paroxetine | 6 (2) | Beclomethasone dipropionate | 18 (6) |
| Infliximab | 91 (5) | Fluvoxamine | 2 (1) | Mometasone furoate / Formoterol fumarate | 13 (4) |
| Azathioprine | 81 (5) |  |  | Fluticasone furoate / Vilanterol | 6 (2) |
| Other systemic steroids | 81 (5) |  |  | Mometasone furoate | 5 (2) |
| Sulfasalazine | 52 (3) |  |  | Ciclesonide | 5 (2) |
| Other antirheumatic medications | 63 (4) |  |  | Salmeterol xinafoate | 3 (1) |

*Note:* The exposures are presented for the combined cohorts of continuers and discontinuers. Exposure to multiple medications within the group was possible. Mothers in the asthma group could also be treated with other asthma medications, including short-acting bronchodilators and montelukast, but not with systemic steroids. SSRIs: Selective Serotonin Reuptake Inhibitors, %: Percent within the exposure group

**Supplementary Table 3**

*Indicators of Goodness of Fit for the Adjusted Analyses Performed With Modified Poisson Regression Models With Robust Covariance in Table 3*

| Exposure Group | Log Likelihood | Pearson Chi Square  Value/dF | Likelihood Ratio  *p** |
| --- | --- | --- | --- |
| Not initiating breastfeeding | | | |
| ARM continuers | -809.3 | 0.97 | < 0.001 |
| SSRI continuers | -564.0 | 0.97 | 0.210 |
| Asthma medication continuers | -564.7 | 0.97 | 0.046 |
| ARM discontinuers | -666.0 | 0.97 | < 0.001 |
| SSRI discontinuers | -530.3 | 0.98 | <0.001 |
| Asthma medication discontinuers | NA | NA | NA |
| Supplementing | | | |
| ARM continuers | -3020.4 | 0.66 | 0.065 |
| SSRI continuers | -2735.8 | 0.66 | 0.011 |
| Asthma medication continuers | -2672.3 | 0.67 | < 0.001 |
| ARM discontinuers | -2837.6 | 0.66 | 0.006 |
| SSRI discontinuers | -2572.7 | 0.67 | 0.242 |
| Asthma medication discontinuers | -2607.1 | 0.67 | 0.003 |

*Note:* ARM: Antirheumatic Medications, SSRI: Selective Serotonin Reuptake Inhibitors, dF: Degrees of freedom

* Significance of the Omnibus test with Likelihood Ratio, of whether the independent variables collectively improve the model over the intercept-only model, *p*<.05 Statistically significant

**Supplementary Table 4**

*Indicators for Goodness of Fit for the Adjusted Cox Regression Models in Table 3*

| Exposure group | Chi Square | dF | *p* |
| --- | --- | --- | --- |
| ARM continuers | 37.55 | 2 | <0.001 |
| SSRI continuers | 19.81 | 2 | <0.001 |
| Asthma medication continuers | 7.00 | 2 | 0.030 |
| ARM discontinuers | 7.97 | 2 | 0.019 |
| SSRI discontinuers | 28.13 | 2 | <0.001 |
| Asthma medication discontinuers | 2.29 | 2 | 0.319 |

*Note:* ARM: Antirheumatic Medications, SSRIs: Selective Serotonin Reuptake Inhibitors, dF: degrees of freedom
